# Epilepsy and Peripheral Irritation

**Published:** 1905-05-13

**Authors:** 


### EPILEPSY AND PERIPHERAL IRRITATION.

Attention was recently drawn in these columns to the multifarious activity of reflex processes, in connection with the treatment of spasmodic asthma by cauterisation of the nasal septum. A striking analogy is reported by Dr. St. Clair Thomson,[1] in which the source of peripheral irritation was adenoid overgrowth in the posterior nasal passages, and the response minor epilepsy.

A child of six had been subject to attacks of petit mal for two years. She was a well-marked mouth-breather with the typical adenoid face, and was, moreover, considerably deaf. A large mass of adenoids was removed by operation, and within a fortnight the mouth-breathing had ceased, but the fits continued for four months, during which time she continued to take bromides. At this time the fits abated and the bromide was presently stopped. At the time of writing this patient was 13 years of age, had had no fits for seven years, and no bromides for six years. The author concludes as follows:—"I have had other cases where adenoids have been removed in epileptic children without the satisfactory result above recorded, but I am prompted to put even one success on record. For there appears to be a general concensus of opinion that the most unsatisfactory cases of epilepsy are those in which the disease commences under 10 years of age." This is an undoubted fact, and the success proportionately gratifying; it serves, moreover, to point the moral that any rule of thumb method, such as blind adherence to bromides, is fatal to the maximum of success in the treatment of epilepsy. It is probably not too much to say that every part and organ of the body is a potential source of epilepsy in a predisposed subject, as are demonstrably the upper air-passages and the alimentary canal, and a comprehensive survey of the bodily functions in each individual case is an essential prelude to the treatment of the disease.

[1] Pract., May 1905.

